# Early and fair COVID-19 outcome risk assessment using robust feature selection

**DOI:** 10.1038/s41598-023-36175-4

**Published:** 2023-11-03

**Authors:** Felipe O. Giuste, Lawrence He, Peter Lais, Wenqi Shi, Yuanda Zhu, Andrew Hornback, Chiche Tsai, Monica Isgut, Blake Anderson, May D. Wang

**Affiliations:** 1 The Wallace H. Coulter Department of Biomedical Engineering, Georgia Institute of Technology and Emory University, Atlanta, GA 30322 USA; 2 School of Electrical and Computer Engineering, Georgia Institute of Technology, Atlanta, GA 30322 USA; 3 School of Computer Science and Engineering, Georgia Institute of Technology, Atlanta, GA 30322 USA; 4 School of Biology, Georgia Institute of Technology, Atlanta, GA 30322 USA; 5 Department of Medicine, Emory University, Atlanta, GA 30322 USA

**Keywords:** Predictive markers, Risk factors, Machine learning, Predictive medicine

## Abstract

Personalized medicine plays an important role in treatment optimization for COVID-19 patient management. Early treatment in patients at high risk of severe complications is vital to prevent death and ventilator use. Predicting COVID-19 clinical outcomes using machine learning may provide a fast and data-driven solution for optimizing patient care by estimating the need for early treatment. In addition, it is essential to accurately predict risk across demographic groups, particularly those underrepresented in existing models. Unfortunately, there is a lack of studies demonstrating the equitable performance of machine learning models across patient demographics. To overcome this existing limitation, we generate a robust machine learning model to predict patient-specific risk of death or ventilator use in COVID-19 positive patients using features available at the time of diagnosis. We establish the value of our solution across patient demographics, including gender and race. In addition, we improve clinical trust in our automated predictions by generating interpretable patient clustering, patient-level clinical feature importance, and global clinical feature importance within our large real-world COVID-19 positive patient dataset. We achieved 89.38% area under receiver operating curve (AUROC) performance for severe outcomes prediction and our robust feature ranking approach identified the presence of dementia as a key indicator for worse patient outcomes. We also demonstrated that our deep-learning clustering approach outperforms traditional clustering in separating patients by severity of outcome based on mutual information performance. Finally, we developed an application for automated and fair patient risk assessment with minimal manual data entry using existing data exchange standards.

## Introduction

COVID-19 testing is now commonplace, and has become a requirement for many public activities. It has been demonstrated that early treatment of COVID-19 positive decreases risk of serious adverse events^1–3^. Nevertheless, a standard approach to triaging patients with positive test results to optimize treatment delivery has not been established. This is in part due to the wide variation in symptom severity among patients and the paucity of recent clinical data on patients available at the time of diagnosis. Therefore, it is crucial to predict severe patient outcomes once a positive test result has been obtained. In addition, it has been demonstrated that COVID-19 healthcare outcomes are disproportionately more devastating in traditionally underserved populations^4^. Understanding how data-driven solutions to healthcare delivery optimization affects these populations is vital to the equitable delivery of potentially life saving care.

To address this ongoing challenge, we generated an interpretable AI workflow to predict individualized risk for death and ventilator use using data available at the time of COVID-19 diagnosis. To support our predictions, we visualize the importance of patient-level features using SHapley Additive exPlanations (SHAP)^5^. In addition, we generate a robust rank of clinical feature importance to provide clinical insights into the factors most influential to catastrophic outcomes. Specifically, we use explainable AI techniques to rank features by their importance across seven different conventional machine learning models and four variants of a deep learning model. A final feature ranking was generated using an average weighted rank of each feature across all models weighted by model performance, measured by area under the receiver operating curve (AUROC).

To further elucidate the factors affecting patient outcomes, we sought to identify the best unsupervised clustering approach to separate patients with severe outcomes from those without. Specifically, we clustered patients using three conventional approaches (agglomerative, K-means, and spectral clustering) and a novel deep learning-based clustering technique. For each conventional clustering method, we generated 2, 3, 5, and 10 clusters with five different feature sets (four deep learning features and the original clinical features). To compare clustering approaches, we used normalized mutual information (NMI) to measure the ability of each approach to separate patients with severe outcomes from non-severe outcomes.

**Figure 1:**
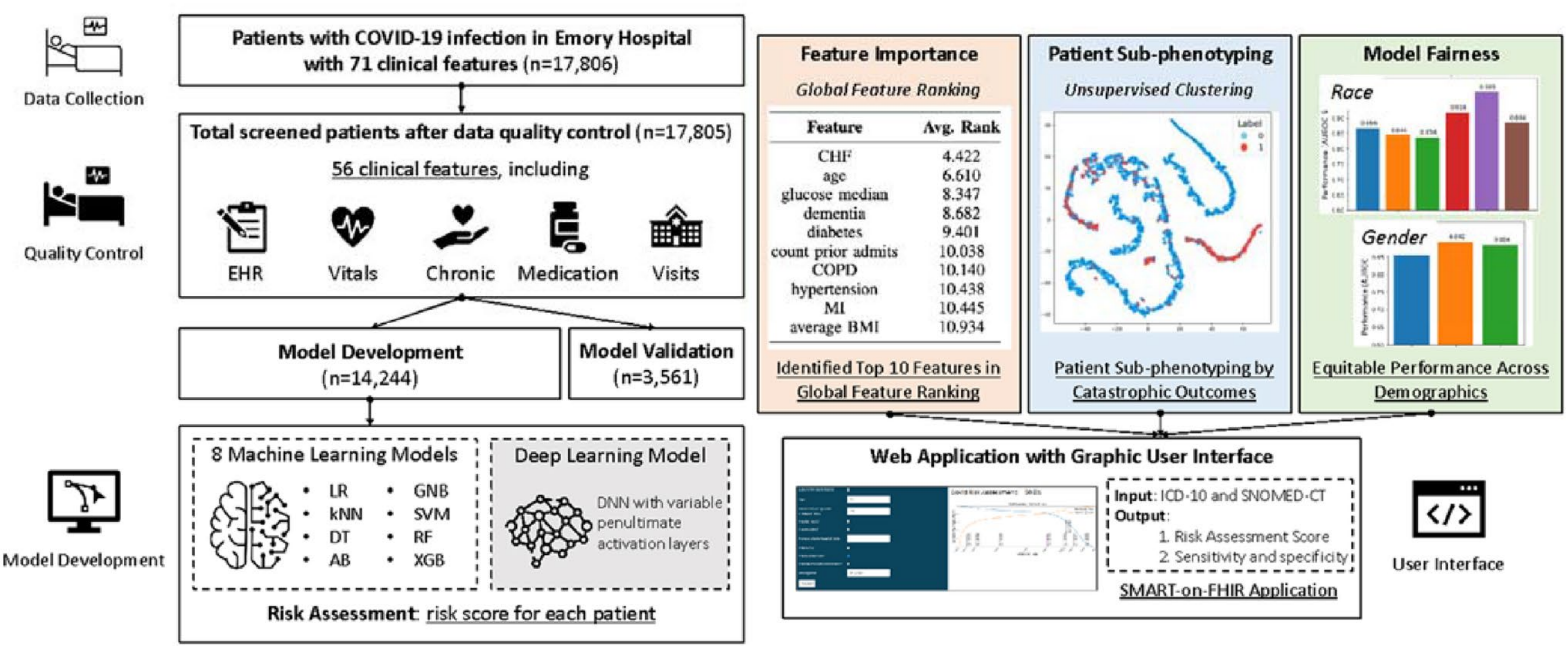
Overview of our approach to generation of explainable clinical decision support tools for clinical outcomes risk prediction.

Finally, we establish the equity of our model across race and sex by comparing differences in performance of our optimized classifier within patient populations in our holdout testing dataset, as shown in Fig. 1. We show that our approach generates accurate and interpretable risk predictions and meaningful clinical insights by leveraging a robust combination of machine learning and explainable AI approaches (Fig. 1). A preliminary version of this work, which established a proof-of-concept of using our deep learning framework for outcomes prediction, has been reported^6^. Our major contributions in this work include:

- We developed an interpretable clinical decision support system for patient risk assessment.
- We demonstrated equitable model performance across communities most severely affected by COVID-19.
- We generated meaningful clinical insights using robust feature importance rankings and clustering approaches to identify novel biomarkers for patient outcomes.
- We facilitated clinical deployment of our optimized model via a user-friendly web application.

## Related works

### Risk prediction

Timely risk assessment of COVID-19 patients can significantly improve the quality of patient care and in-hospital resource allocation^7^. Recent studies have leveraged machine learning to derive and validate risk prediction algorithms via electronic health records (EHRs) in order to estimate the risk of COVID-19-related adverse events, such as ICU readmission and mortality.

Yan et al.^8^ proposed conventional machine learning tools to predict 10-day mortality based on the blood sample data of 485 infected patients in order to support decision-making and logistical planning in healthcare systems during pandemics. The machine learning model achieved greater than 90% accuracy, and several positive results from external validation^9–11^ demonstrated the effectiveness of the proposed model in early and accurate risk assessment for COVID-19 patients. Kuanr et al.^12^ used a patient similarity-based approach to develop a patient recommendation system. Fu et al.^13^ proposed a risk prediction model using logistic regression based on laboratory findings for the early identification of high-risk hospital admission patients and achieved an AUROC of 84% during external validation. In addition, Barda et al.^14^ proposed a hybrid methodology to develop a baseline severe respiratory infection risk prediction model and a post-processing algorithm to calibrate the predictions to reported COVID-19 mortality risk using population-level data. Similarly, several recent studies^15–30^ also took advantage of conventional machine learning methods (e.g., Logistic Regression, Support Vector Machine, XGBoost, Decision Tree, and Random Forest) to develop a machine learning based early warning system enabling mortality or hospital admission risk prediction for COVID-19 patients.

Existing studies have demonstrated that early warning risk prediction models play a significant role in the allocation of scarce hospital resources^8,13–32^; however, there are still several limitations preventing the widespread adoption of risk prediction models in clinical practice. First, compared to conventional machine learning algorithms, fewer studies^15,18,28,31–33^ have been conducted on COVID-19 risk prediction based on deep learning methods, which have the potential to achieve better performance due to their enhanced capability to perform representation learning. Second, model transparency is one of the most significant obstacles preventing clinicians from comprehending and implementing black box models (e.g., XGBoost, neural networks) in clinical practice^34^. Few existing studies tried to interpret prediction models by identifying potential important clinical features using tree-based^8,32^ or SHAP feature importance^14,21,27,33^. In addition, none of the aforementioned studies have considered the issue of model fairness to achieve equitable model performance across the most severely affected communities. Finally, without integrating a prediction model into a functional web application with a user interface, it is extremely difficult to promote the adoption of the technology among non-technical users. Few existing studies^35,36^ have taken user interface into consideration to provide real-time progression prediction and risk calculation for policy decision-making and intervention guidance.

### Model fairness

During the COVID-19 pandemic, AI-enabled clinical decision support became an integral part of the diagnosis, triage, and treatment processes. Gender, racial, and other ethnic disparities in healthcare have been well-documented in terms of COVID-19 infection, hospital admissions, and in-hospital outcomes^37^.

According to COVID-19 inequity and disparities studies, different gender groups have different levels of risk associated with COVID-19 infection and mortality, with males having a higher COVID-19 death rate across all age groups^38^. Existing studies have also highlighted racial disparities through the non-representative morbidity rate, hospitalization risk, and mortality rates among Black and Latinx populations^39–41^. In addition, African Americans are at a greater risk for COVID-19 due to the higher prevalence of comorbidities, such as diabetes and hypertension^39^. Furthermore, Black people and other minority groups are more susceptible to both the acute and long-term effects of COVID-19 due to a lack of access to adequate healthcare services^42^. As a result, identifying gender- and race-specific clinical bio-markers is important for the development of risk prediction tools to combat systemic healthcare disparities for racial and ethnic minorities^43^.

Consistent model performance across demographic populations is important for optimal and fair patient care decision making. However, it is challenging to achieve robust model performance in underrepresented populations due to the large data requirements of cutting-edge models. To solve this problem, Yang et al.^44^ introduced an adversarial training framework for rapid COVID-19 diagnosis to mitigate demographic biases that acquired from data collection and magnified during model development. In risk prediction for other diseases, Do et al.^45^ proposed a joint fairness model based on logistic regression models for binary outcomes that estimates group-specific classifiers with a joint modeling objective function integrated fairness criteria. In addition, Pfohl et al.^46^ established a counterfactual fairness reasoning model that extends the group fairness criterion of equalized odds to provide a trade-off between maintaining fairness and performance.

During the data collection phase, our patient cohort contains a robust representation of patients from the Atlanta metropolitan area in order to promote model equity and reduce disparities in COVID-19 risk prediction. During the stage of model development, we examine the impact of significant characteristics on the output of our models for potential bio-maker exploration, including those associated with sex and race, as well as clinical characteristics of particular importance. In addition, we analyze biases in additional features that may have contributed to the effect of these significant features in order to provide a fair machine learning model for COVID-19 risk prediction. Our research on individual equalized risk prediction could also be extended to future disease risk prediction beyond COVID-19 in order to achieve a robust and consistent clinical decision support.

## Materials and methods

### Data description and preprocessing

Our dataset consisted of de-identified data from 17,806 COVID-19-positive patients containing 71 clinical features from across the Emory University Hospital System sites in Atlanta, Georgia, USA (Table 1). Prior to using the data for model training, we conducted quality control on the dataset. We first removed 11 discrete features with a non-zero value in less than 1% of all patients to eliminate any noise that may have been introduced in the data by rare, or incorrectly entered, medications or conditions (i.e. outliers). We then removed 4 features that were missing in more than 75% of all patients, because these features may not be reliably filled in with data imputation. Among the remaining features, we normalized all continuous features followed by imputation of missing feature values (12.6% of remaining values) using K-nearest neighbors imputation (with K = 5). One patient was removed because they were missing over 40% of their features (a clear outlier on visual analysis). A 80/20 split at the patient level was performed to create training and testing cohorts. The resulting dataset consisted of 17,805 patients containing 56 clinical features including: 17 drug categories, 17 vitals and labs, 14 chronic conditions, 6 demographic features, and 2 prior hospital visit features (number of prior ER visits and number of prior hospital admissions). The primary outcome variable was any catastrophic outcome, defined as the use of a ventilator or death within 90 days after positive COVID-19 testing.

**Table 1:** Patient demographics.

Demographics	Patients (%)	Death within 90 days (%)	Ventilator (%)	Any catastrophic (%)
Sex				
Male	7695 (43.2)	308 (55.9)	562 (57.2)	659 (56.4)
Not male	10,111 (56.8)	243 (44.1)	420 (42.8)	510 (43.6)
Race				
African American	7367 (41.4)	565 (57.5)	262 (47.5)	640 (54.7)
Asian	506 (2.8)	29 (3.0)	16 (2.9)	35 (3.0)
Caucasian	5050 (28.4)	279 (28.4)	215 (39.0)	373 (31.9)
Hispanic	964 (5.4)	67 (6.8)	28 (5.1)	69 (5.9)
Other/unknown	4360 (24.5)	66 (6.7)	42 (7.6)	77 (6.6)
Total	17,806	551 (3.1)	982 (5.5)	1169 (6.6)

### Ethical approval and informed consent

The Institutional Review Board (IRB) of Emory University, Atlanta, Georgia, USA granted the ethics approval in 2020 (Protocol STUDY00001408). All experiments were performed in accordance with relevant guidelines and regulations; informed consent was obtained from all participants. All research data are de-identified and securely stored. Data access is limited to approved study personnel.

### Models

Risk scores within the range [0,1] indicating a person’s susceptibility to catastrophic outcomes from COVID-19 infection were first calculated for all patients using seven conventional learners: Logistic Regression, K-Nearest Neighbors (KNN), AdaBoost^47^, Gaussian Naive Bayes, Support Vector Classifier (SVC)^48^, Random Forest^49^, and XGBoost^50^. Specifically, we performed hyperparameter tuning independently for each model using grid-search and five-fold cross-validation on the training dataset. Throughout this process, we developed a total of 216 variations of the aforementioned conventional models. The best hyperparameter set for each model was chosen as the set producing the highest mean AUROC score across the five-folds. Hyperparameter tuned models were used for all subsequent analyses.

Deep learning models were trained on the training dataset. Giuste et al. demonstrated a process of optimizing a neural network architecture including the number of fully connected layers, the depth of each layer, and the intensity of each dropout layer^6^. Thus, we used the developed common core architecture in our deep learning classifier models. Expanding upon this work, we developed and tested 44 different variations of this deep learning model framework to further optimize two hyperparameters using the training dataset: the activation function for the penultimate layer and the depth of the penultimate layer. Specifically, activation functions for the penultimate layer included ReLU, Sigmoid, Softmax, and Gumbel-Softmax; tested sizes for the depth of the penultimate layer included 2, 3, 4, 5, 8, 10, 15, 20, 30, 50, and 100 neurons. Optimal values for the core architecture, such as overall model depth and sizes for layers other than the penultimate layer, were determined by analyzing the model performance (AUROC)^6^. Early stopping with patience of 100 was used to stop model training when testing AUROC failed to improve after 100 epochs. Class imbalance effects were minimized by balancing each training epoch to ensure the model would be exposed to the same amount of data from each class. Specifically, during each epoch, we trained the models on all data points from the minority class (catastrophic outcome) and a random sample of observations from the majority class (no catastrophic outcome). The size of this random sample was equal to the size of the minority class. We utilized this technique to prevent our models from developing a bias in favor of the majority class.

### Feature importance

SHAP feature importance values were calculated for each conventional learner to identify the clinical features that were most influential in generating risk score predictions^5^. Average SHAP values across training and validation patients were used to rank the features by importance for each conventional and deep learning model variant. We obtained the feature rankings for each conventional model. These feature rankings were weighted by model performance and averaged across models to obtain a list of the top ten most important features across conventional models and deep model variants.

The original feature rankings of all high performing conventional learners and deep learners were also weighted by model performance and averaged to generate a third table demonstrating the top ten most important features across conventional and deep learning models.

### Clustering

After training, the features generated in the penultimate layer of each deep learning model were obtained and used for clustering. The generated deep features were of special interest due to their potential to separate patients into groups of different (high vs. low) risk scores more distinctly than existing clinical features. We therefore compared the distribution of patients qualitatively and quantitatively using clinical and deep feature spaces to identify meaningful patient clusters to generate clinical insights.

We first sought to qualitatively analyze how patients were distributed in the clinical feature space as compared to the deep feature space. We did so using T-distributed Stochastic Neighbor Embedding (t-SNE) plots^51^, which provides a lower-dimensional projection of high-dimensional feature spaces for visualization of observation clusters. We first generated a baseline t-SNE plot of points using only the preprocessed clinical feature space, followed by several additional t-SNE visualizations of the deep feature spaces for each deep learner. Points in each plot were colored according to whether their corresponding patients had a low or high risk of catastrophic COVID-19-related outcome based on ground-truth labels. This approach allows the qualitative analysis of potential clusters and their relationships with our primary outcome of interest (i.e., catastrophic outcomes).

Following t-SNE plot generation, we sought to quantitatively rank the quality of deep feature clusters in relation to clinical feature clusters. To generate and compare sets of clinical feature clusters, we used three clustering algorithms including K-Means, Agglomerative, and Spectral clustering with number of clusters (K) equal to 2, 3, 5, and 10. To generate deep feature clusters, we used the same three clustering algorithms on the feature spaces corresponding to three of our four deep learning variants (ReLU, Sigmoid, and Softmax). Our Gumbel-Softmax approach automatically assigns each patient to a single cluster, where each cluster is represented as a dimension of the deep learning feature space. Therefore, this approach does not require independent feature generation and clustering approaches. Altogether, 37 sets of deep feature clusters were generated in total.

To quantify clustering performance, we calculated the normalized mutual information (NMI)^52^ of each set of clusters with the known feature indicating catastrophic outcome. Higher NMI scores for a clustering approach corresponds to better separation of patients based on this outcome of interest. A high NMI of clusters with our primary outcome suggests effective separation of patients by meaningful clinical endpoints, lending credibility to our unsupervised approach. We also compared NMI scores of the deep feature clusters to MI scores of the clinical feature clusters to test the hypothesis that the utilization of our novel deep learning feature spaces may better separate low- and high-risk patients compared with clinical features.

Having ranked all cluster sets based on NMI score, we then viewed the five best-performing clustering results in greater detail by generating bar plots that illustrated how low- and high-risk patients were segregated within the clusters. We compared our observations to similar bar plots illustrating patient distributions within the five best-performing clinical feature clusters, visually comparing the compositions of cluster sets to make observations concerning the quality of deep and clinical feature clusters.

### Model performance equity across demographics

We sought to confirm that our models achieved similar levels of performance across different sexual and racial groups. We first analyzed our XGBoost classifier trained on the entire feature set. To analyze the differences between sex groups, we calculated the performance of the model using the AUROC score for males and females. To analyze the differences between race groups, we calculated the performance of the model using the AUROC score for African Americans, Asians, Caucasians, Hispanics, and unlabeled patients. We performed this same process for the XGBoost classifier trained on just the top ten features determined previously. We also performed this same process for the optimized deep learner (ReLU variant) trained on the full feature set and the optimized deep learner trained on just the top ten features. This approach allowed us to determine if decreasing the number of input features during model training would affect the bias of model performance.

### Final model evaluation on holdout set

Finally, we tested our ReLU model variant and XGBoost model on our holdout (e.i. test) dataset (20% of the preprocessed dataset). We also compared the test-set performance of our trained models on the full feature set as well as those trained on just the top ten feature set. The feature subsets were determined previously using training data only. The degree of importance attributed to the top features was then investigated. We created eleven subsets of the original processed dataset. The first feature subset included only the top ten most important features. Each of the other ten subsets included the top-10 features, iteratively excluding one of the top ten features (e.g. all top-10 features except dementia), for a total of 9 features each. The conventional and deep learners were retrained using the previously outlined procedures with each feature subset. AUROC values were collected and compared to the original AUROC values. A Kruskal–Wallis H-test test was used to determine whether each feature subset performed significantly differently from the original processed dataset using results obtained from all models (each observation was the test-set AUROC of a single trained model).

## Results

### Risk prediction

**Figure 2:**
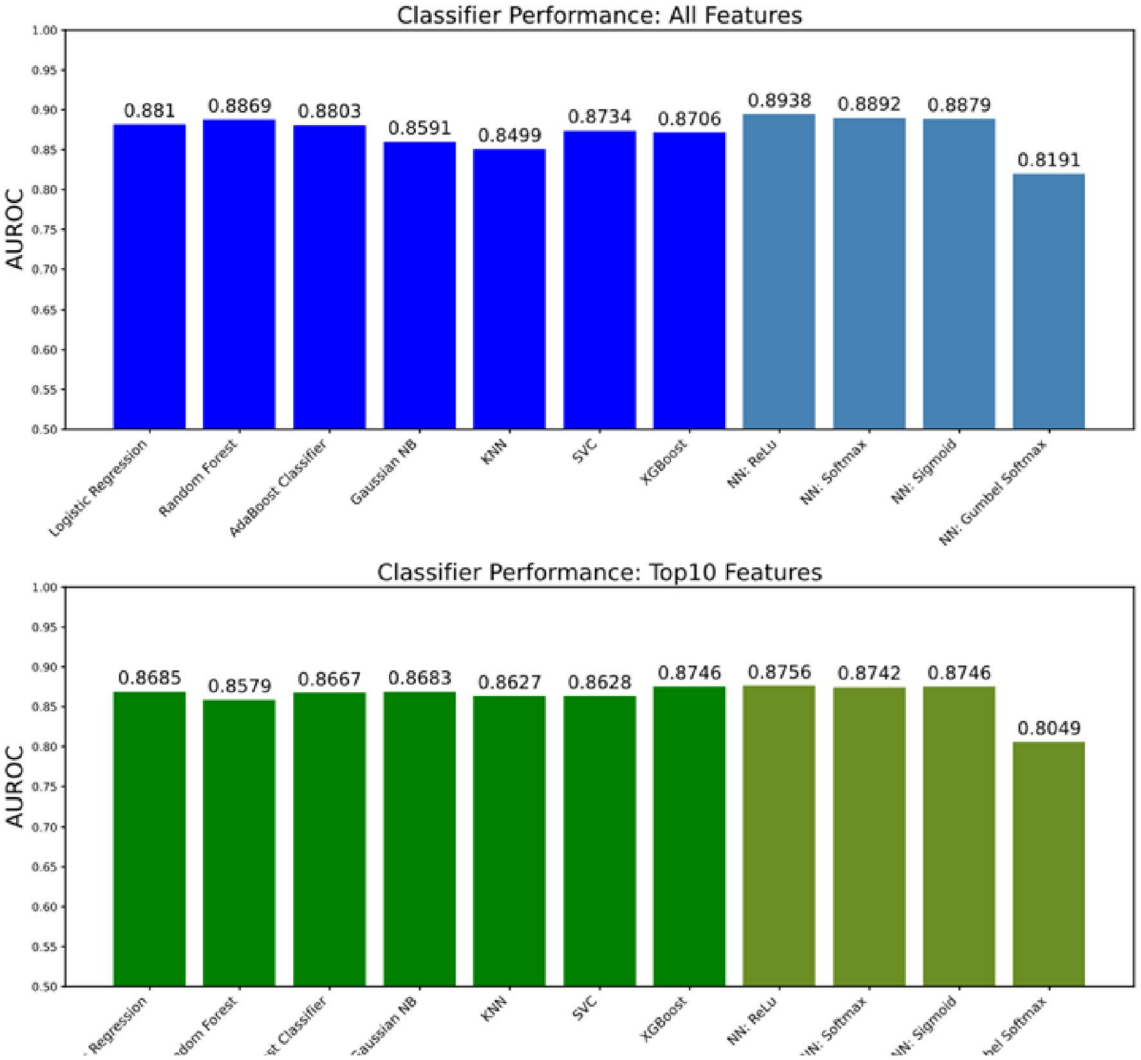
Model performance (AUROC) from models trained on just the top 10 features compared with models trained on all features. Performance metrics for the 11 trained classifiers were generated on the same test set, which was not seen during training. We show that the performance of the 7 conventional learning approaches is comparable with that of the 4 deep learning architectures. Top: Performance of models trained on all features. Bottom: Performance of models trained on the top 10 features. K-Nearest Neighbors (KNN), Support Vector Classifier (SVC), Neural Network (NN). Blue and green bars represent conventional models trained on all features or just the top 10 features, respectively. Light blue and light green bars represent deep learning models trained on all features or just the top 10 features, respectively.

**Figure 3:**
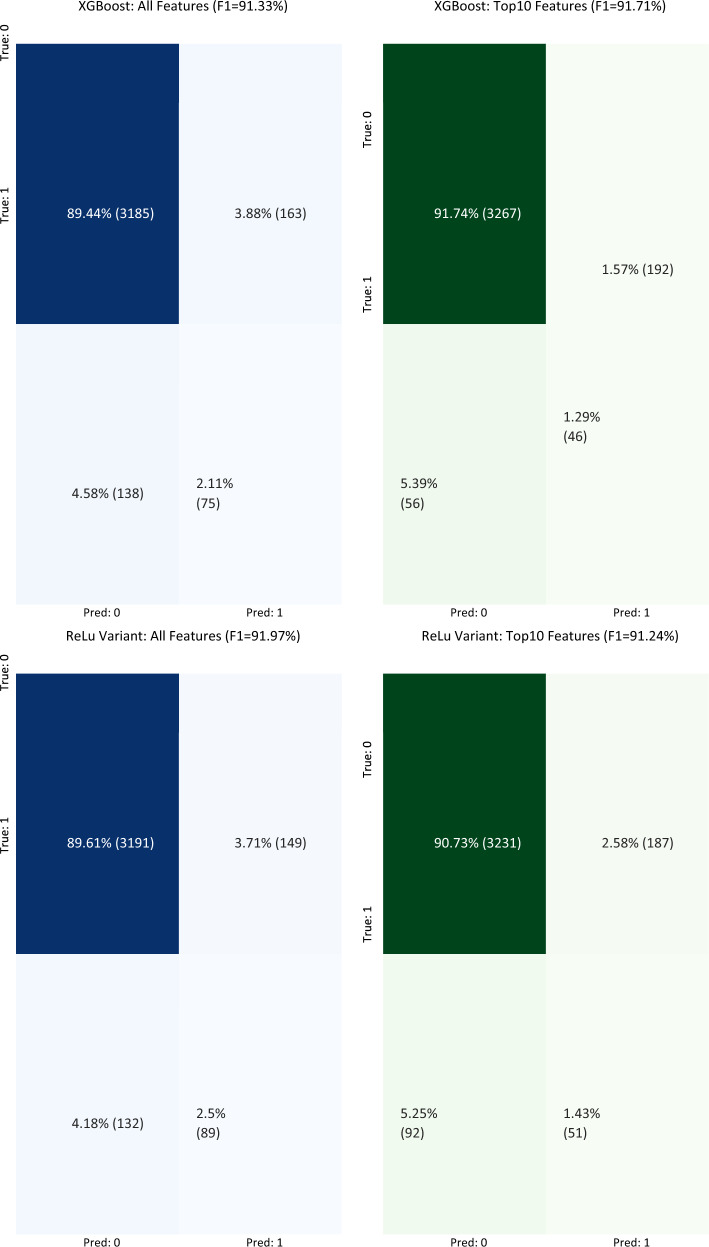
Confusion matrices illustrating the performance of our XGBoost and ReLU neural network models trained on either all features or just the top 10 features. (**A**–**D**) The threshold used to binarize model risk estimates (expressed as probabilities) was determined for each model to maximize the weighted averaged F1 score on the training dataset. The performance scores on the testing (holdout) dataset for the four models were: 91.33% (XGBoost, all features), 91.71% (XGBoost, top 10 features), 91.97% (ReLU, all features), and 91.24% (ReLU, top 10 features). The numbers in parentheses represent the number of test set observations within each category. Blue matrices represent models trained on all features, and green for models trained on just the top 10 important features.

Out of our best 7 conventional conventional learning models tested on the testing dataset, XGBoost, Random Forest, AdaBoost, and SVC all obtained AUROC scores over 87% (Fig. 2) and weighted averaged F1 scores over 90% (Fig. 3). The worst performing conventional model was KNN with an AUROC of 84.99%. These positive results are consistent with previously published works using these models on clinical decision support tasks^15,16,18,19,21,23,24,30^. Our 4 deep learning model variants, which differ by their penultimate activation function, performed similarly to conventional models, with the exception of the Gumbel Softmax variant. This was not unexpected as the Gumbel Softmax activation layer one-hot encodes the data, which greatly decreases the information available for the final classification layer. Despite this significant reduction in granularity, the Gumbel Softmax variant still achieved AUROC of 81.91% while automatically assigning each patient to a mutually exclusive cluster. To the best of our knowledge, this is the first work using Gumbel Softmax to enable deep-learning clustering and classification within the same model.

#### Test performance

XGBoost and our ReLU neural network were chosen as representative conventional and deep learning models (respectively) for testing on our holdout dataset (Fig. 2). In addition, we trained our two models using just the top 10 global features (see Table 2) to understand the impact of feature reduction on model performance. We show that the AUROC of our conventional and deep models are similar (AUROC of 88.7% and 88.8%, respectively) when trained on all available features in the training dataset and applied to our holdout dataset. In addition, we note that training our two models using our identified top 10 features reduced performance by less than 2% for both models when applied to the holdout dataset. This is significant because clinical models with many features may pose a significant burden on clinicians if manual data entry is required. Many features take time to be found in the patient’s electronic healthcare records, and the fewer features required to obtain a reliable risk assessment, the more time the clinician has to spend on shared decision making with the patient. This is especially important in urgent care settings where decisions must be made quickly and reliably.

We examine the sensitivity and specificity of our deep learning (ReLU) model trained on only the top 10 clinical features across model risk prediction probabilities to allow end-users to customize threshold for their specific clinical setting (e.g., urgent care vs. asymptomatic testing). The sensitivity and specificity of our model are identical (both 81.93%) when predicted risk for catastrophic outcome is equal to 64.2%. If additional sensitivity is required, as may be the case when treatment is cheap and effective, a risk threshold of 28.8% may be used to achieve a sensitivity of 90.34% and specificity of 71.14%. We achieved similar AUROC score to those reported in peer-reviewed literature^53–55^. Estiri et al.^53^ reported a mean AUROC score of 0.898 using generalized linear model with gradient boosting on mortality prediction with electronic health records of 16,709 COVID-19 patients. Sottile et al.^54^ presented a stacked generalization model for mortality prediction on COVID-19 patients using EHR data, achieving 0.94 AUROC score, outperforming the baseline models based on Charlson Comorbidity Index (0.72 AUROC score) and sequential organ failure assessment (SOFA) (0.90 AUROC score). Therefore, our best model achieves state-of-the-art results for risk analysis.

### Global feature ranking

The presence of hypertension, age, congestive heart failure (CHF), diabetes, and dementia are highly ranked after calculating their weighted average rank across conventional models. It has been well established that there exists a strong relationship between kidney disease and CHF^56^. The presence of these features likely demonstrate a patient profile which is already suffering from chronic disease, which results in an increased COVID-19 burden on their health. Along with CHF, dementia, and kidney disease are also top important features within our deep learners. It may be interesting to note that our explainable AI approach identified count of prior admissions as an important feature within deep learning models, but not in our conventional learners. Count of prior admits may be increased in patients with chronic disease, which would be consistent with the chronic diseases identified by both models.

We were determined to understand the clinical biomarkers most important for determining catastrophic outcome risk. To achieve this goal, we used SHAP to examine the rank of each clinical feature within each model, and across model subsets (e.g., conventional and deep models). As each model generates similar, but different, feature ranks, we calculated the weighted average rank of each feature, using model performance (measured by AUROC) as the weight (Table 2). We show that the presence of dementia is a consistently important clinical feature for predicting catastrophic outcome. This insight becomes clearer after comparing the average ranks of all features. The top three important features across all models: CHF, age, and high glucose, are consistent with prior literature on catastrophic COVID-19 outcome risk^57^. Results of our statistical analysis of models trained on feature subsets indicate that using top-10 features does not significantly decrease model performance (p = 0.1228), yet removing additional top-10 features, including CHF, dementia, count of prior admits, and MI, demonstrate significantly reduced performance (p = 0.0197, 0.0138, 0.0328, and 0.0235, respectively). These results supports the use of a much reduced input feature space to facilitate clinical integration by reducing the burden of searching for features within patients’ EHRs and manual data entry (e.g. finding and entering 10 features is much more convenient than 56 features spread across EHR locations and patient visit dates). Many of the features included within the top-10 rankings do not require additional blood tests or imaging. Glucose measurements can be quickly achieved using finger pricks, thus avoiding any delay in patient triage and treatment due to obtaining additional data.

State-of-the-art works used different approaches to identify the top-ranking features in risk prediction. Estiri et al.^53^ used odds ratios with interquartile ranges to estimate the feature importance relative to mortality prediction. Age, several respiratory diseases, and cardiovascular diseases were identified as the top features associated with patient outcome. Similarly, Clark-Boucher et al.^58^ used Firth bias-corrected odds ratios in logistic regression model to report patient demographic and disease-related survey variables as top ranking features. Many of our top-ranking features are reinforced in these literature including patient demographics (e.g. age), cardiovascular disease (e.g., CHF and MI), and respiratory disease (e.g. COPD).

**Table 2:** Weighted average feature ranks across model type.

Conventional learners		Deep learners		All learners	
Feature	Avg. rank	Feature	Avg. rank	Feature	Avg. rank
Glucose median	5.116	CHF	1.000	CHF	4.422
Hypertension	5.142	Count prior admits	2.236	Age	6.610
Age	6.273	MI	3.528	Glucose median	8.347
CHF	6.360	Dementia	4.517	Dementia	8.682
GFR last	9.724	Stroke	5.485	Diabetes	9.401
Diabetes	10.047	Kidney disease	6.025	Count prior admits	10.038
COPD	10.769	Age	7.205	COPD	10.140
Dementia	11.040	Diabetes	8.260	Hypertension	10.438
Average BMI	11.603	COPD	9.027	MI	10.445
Glucose last	12.434	Average BMI	9.751	Average BMI	10.934

### Patient subphenotyping

**Figure 4:**
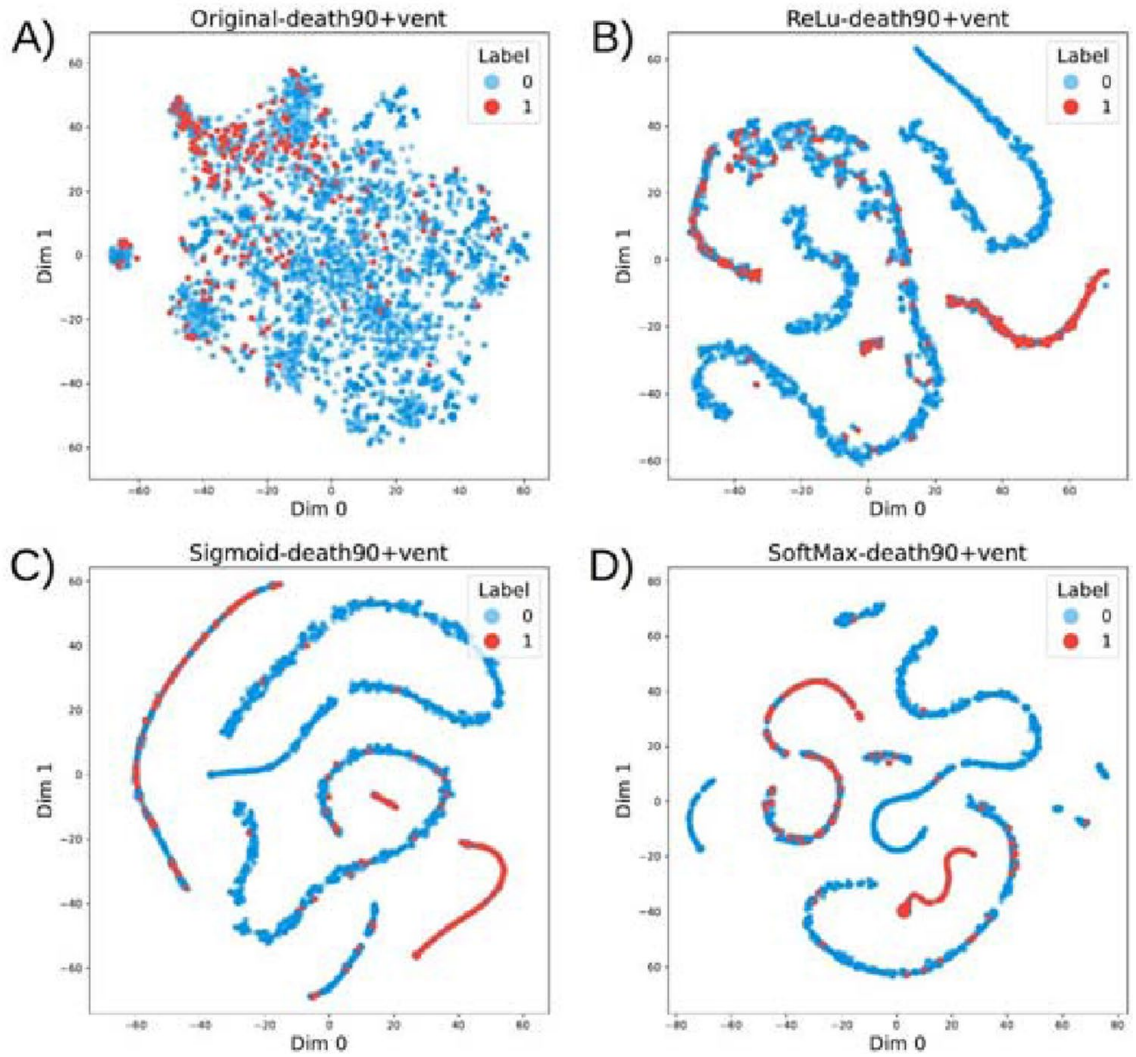
Patients without catastrophic outcomes (blue) and patients with catastrophic outcomes (red) are more clearly separated after t-SNE of patients using deep learning features. Patient outcome was not a feature used during clustering. (**A**) Original clinical features do not clearly separate patients by outcome. (**B**–**D**) Deep learning features (ReLU, Sigmoid, and Softmax variants, respectively) successfully identify patient subgroups based on qualitative analysis.

Early differentiation of patients into subphenotypes based on their likelihood of suffering catastrophic outcomes may provide insights into the clinical spectrum of disease presentation. Visual inspection of patient similarities using the original clinical features available at the time of diagnosis results in poorly differentiated sub-populations using t-SNE visual inspection (Fig. 4A). There is an increased concentration of patients suffering catastrophic outcomes within the top-left area after t-SNE transformation of patient features, which we hypothesized could be further isolated from the general population using alternative clustering approaches. We show that clustering of patients using features obtained by deep models produces qualitatively improved patient separation by our outcome of interest (catastrophic outcomes) (Fig. 4B–D). Patients suffering from catastrophic outcomes (not a feature used to generate clusters) were colored in orange after t-SNE embedding to aid in visualization of qualitative clusters. Our deep learning models (Softmax, Sigmoid, and ReLU variants) generated patient features which result in more enriched groups of catastrophic patients (Fig. 4B–D), as compared with using clinical features directly for clustering (Fig. 4A).

**Figure 5:**
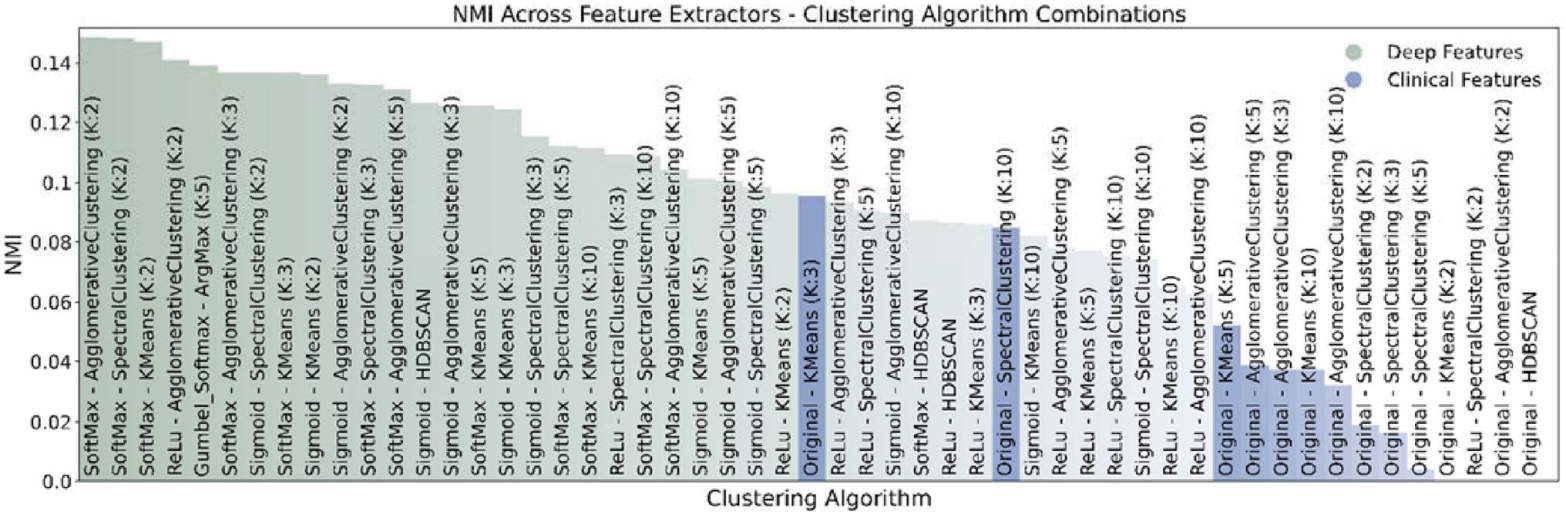
Quantitative comparison of clustering approaches via NMI analysis using deep features (green) or original clinical features (blue). Mutual information is generated by comparing overlap between cluster labels with patients suffering catastrophic outcomes to measure clustering effectiveness. Gumbel-Softmax model variant requires no additional clustering method to assign patients to clusters, and is within the top-5 clustering approaches.

Quantitative comparison of clustering approaches demonstrates that deep features produce better separation of patients by our outcome of interest compared with clinical features alone (Fig. 5). This result is consistent with our understanding of the deep learning training process, as deep features were generated during the patient classification task, and therefore would serve as an improved patient descriptor for separation of patients by outcome compared with the input model features (clinical features). In addition, our proposed Gumbel-Softmax model variant performed within the top-5 of all other approaches without the need for post-hoc clustering of deep features. More specifically, our Gumbel-Softmax model variant learns patient clusters directly at the time of classification, and uses the cluster assignments themselves to classify patients by outcome. These results support the use of our deep learning-based clustering approach for patient classification and clustering within a single model. The largest cluster within all top-5 models contained less than 2% prevalence of catastrophic outcomes, compared with over 20% prevalence within the catastrophic outcome-enriched clusters. It is important to note that the prevalence of catastrophic outcome in our population was 6.6% (1169 out of 17806 in the original dataset).

This significant enrichment of patients with catastrophic outcomes within a single cluster demonstrates the potential use of these clusters for patient triage. In addition, the three top-5 clustering approaches using Softmax variant features assigned patients into much smaller clusters with much lower prevalence of catastrophic outcomes compared with the original population. Patients within these catastrophic outcome-enriched clusters may be placed higher on the priority list for aggressive early treatment to maximize patient outcomes. In addition, patients within clusters with significantly lower percentage of catastrophic outcomes may be treated less aggressively to minimize patient discomfort and healthcare costs. It is interesting to note that our Gumbel-Softmax model variant assigned all but 14 patients to only two clusters, despite having the potential to separate patients into 5 clusters. This feature of the model may be valuable if the number of true clusters within the dataset are unknown. In this case, an upper-bound may be provided to allow the model to chose the appropriate number of final clusters. Future works to establish the robustness of this approach may be conducted prior to clinical integration.

### Equitable model performance

**Figure 6:**
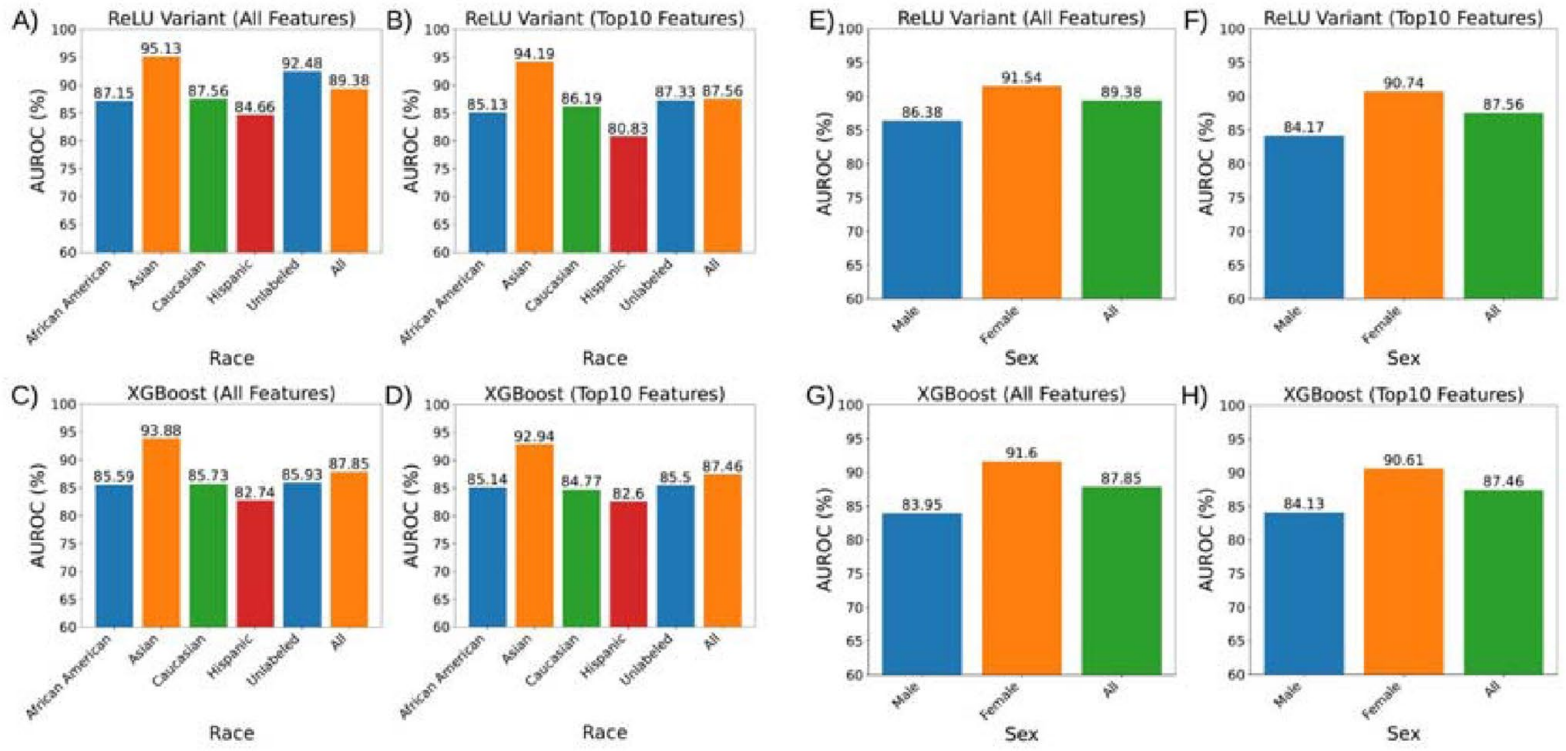
Our conventional and deep learning models perform consistently above 80% AUROC across patient race and gender, even after feature reduction. Performance was highest in those patients identifying as Asian and those which were unlabeled, despite their low representation within our training and holdout (testing) datasets. (**A**) Deep learning ReLU variant performance trained on all clinical features. (**B**) ReLU variant trained on just the top 10 clinical features. (**C**) XGBoost model trained on all features. (**D**) XGBoost model trained on the top 10 clinical features. (**E**) Deep learning ReLU variant performance trained on all clinical features. (**F**) ReLU variant trained on just the top 10 clinical features. (**G**) XGBoost model trained on all features. H) XGBoost model trained on the top 10 clinical features.

Consistent model performance across demographic populations is vital to ensure optimal decision making during patient care. Unfortunately, obtaining robust model performance within underrepresented populations is difficult due to the large data requirements of state-of-art models. Fortunately, our dataset contains a robust representation of patients within the Atlanta metropolitan area. Our top models perform consistently across racial categories, as encoded within the electronic healthcare record system (Fig. 6). In addition, our model performance remains consistent even when trained on only the top 10 clinical features, which does not include race. This supports the application of our model to obtain patient risk for catastrophic outcomes across racial demographics. It is also interesting to note that performance is highest in those patients with Asian or unknown (unlabeled) race, two groups which contain minimal representation within our dataset. The cause of this pattern may be elucidated in future studies which include more granular racial identities. Our top models perform consistently across sex categories, as encoded within the electronic healthcare record system (Fig. 6). In addition, our model performance remains consistent even when trained on only the top 10 clinical features, which does not include sex as a feature. This consistency in performance across sex supports the application of our model to obtain patient risk for catastrophic outcomes across available demographics.

### Application

**Figure 7:**
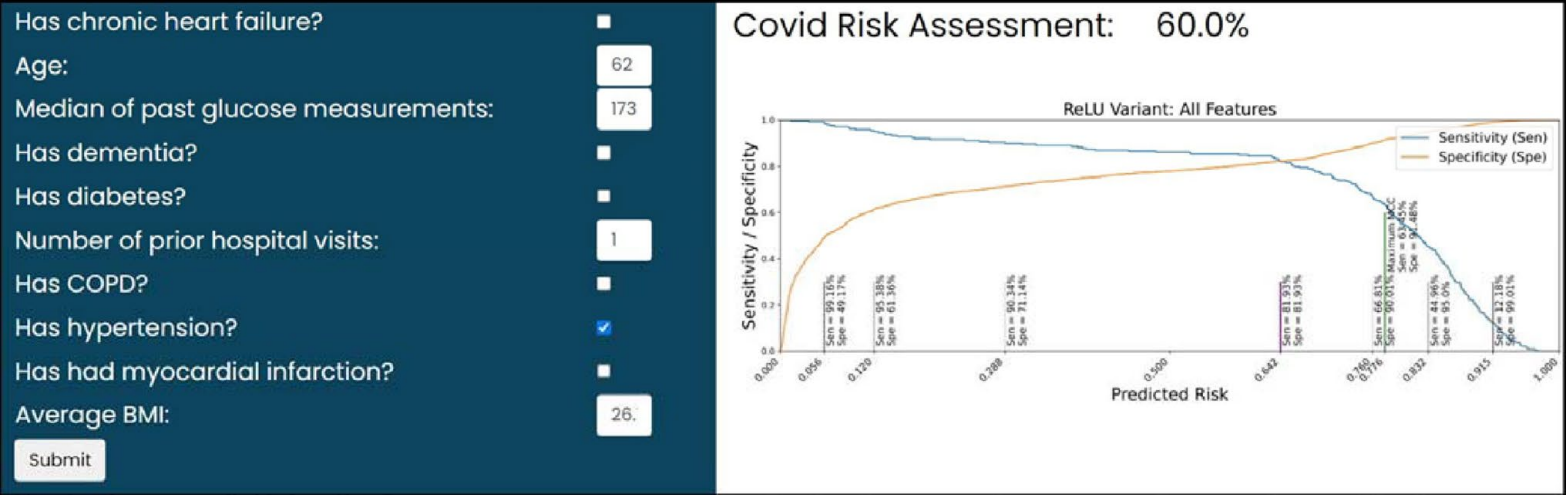
Our web application shows the COVID-19 risk assessment score based on our top ten identified clinical features. Values are automatically filled by accessing patient’s electronic health records using the FHIR data exchange standard. Expert validation of clinical values allows confirmation of correct model input. A plot of model sensitivity and specificity across generated risk scores facilitates clinical interpretation of generated patient risk.

To enhance the real-world clinical impact of our work, we developed a user-friendly web application for automated patient risk assessment based on our findings. Our application is built using the Fast Healthcare Interoperability Resources (FHIR) standard and complimentary SMART-on-FHIR technology, an open-source framework that provides secure access to EHRs. FHIR enables quick and efficient exchange of healthcare data based on modern web information exchange standards^59^. Once a healthcare provider logs in securely and selects a patient within the EHR system, our application uses ICD-10 (International Classification of Diseases, Tenth Revision) and SNOMED-CT (Systematized Nomenclature of Medicine - Clinical Terms) codes to automatically fill in the patient’s clinical features including: dementia, CHF, kidney disease, and COPD status; and whether or not the patient has had a prior myocardial infarction or stroke. ICD-10 and SNOMED-CT are both widely used standards in healthcare for classifying and coding conditions, diagnoses, symptoms, etc., in a systematic manner. The number of prior hospital visits and gender are also automatically extracted. This approach leverages standardized data resources further minimizing data entry time requirements. If any value is unable to be retrieved from the appropriate FHIR resource, the provider can perform manual selection. Once the information is populated, the data is automatically standardized by the application and submitted to our best model (deep learning, ReLU variant) which returns a risk assessment score (Fig. 7).

## Discussion

In this work, we demonstrate an automated approach to conducting patient risk assessment using AI which generates data-driven clinical insights consistent with published clinical and epidemiological research. Specifically, we developed a clinical decision support system which achieves state-of-art performance of 89.38% AUROC early patient risk assessment. In addition, we demonstrated equitable model performance across demographic populations. To improve model interpretation, we generated feature importance rankings and patient clustering to identify robust biomarkers for risk assessment. Lastly, we encouraged clinical testing of our work by reducing model feature requirements and facilitating electronic healthcare record system integration of a user-friendly web application. This work demonstrates the ability of AI to generate clinical insights early in the course of disease for optimizing patient treatment while encouraging usage and trust in clinical end-users.

Our finding of dementia as a significant independent biomarker for severe COVID-19 clinical outcomes demonstrates the utility of our workflow in identifying data-driven patterns within clinical outcomes predictions tasks not previously demonstrated in similar prior work. We demonstrate that this significant effect is due to the presence of dementia, and not due to the association of dementia with age using both SHAP and ablation studies to support this finding. In addition, we elucidate demographic-specific feature contributions to patient-level risks to support the use of our approach where establishing trust in model performance across demographics is vital. Known limitations of our work include the use of data from a single metropolitan region. Although our results demonstrate consistent performance across demographic groups within our cohort, further testing of our approach within individual hospital systems is necessary prior to integration within clinical workflows with significantly different demographic representation.

Future work will focus on expanding our test dataset to include data from additional hospital systems. We will seek to better understand if our approach to generating clinical biomarkers identifies similar patterns across sites and additional demographic groups, or if additional insight may be gained on the relationship of clinical features and disease outcomes. We will also test our approach within other clinical tasks requiring reliable risk prediction early in the disease course (e.g., sepsis and heart disease). We believe this work may establish a consistent approach to clinical decision support tool generation by maximizing interpretability and clinical utility.

## Data Availability

The datasets generated and/or analysed during the current study are not publicly available due to Protected Health Information restrictions applying to the availability of the clinical data, which were used under IRB approval for use only in the current study, but are available from the corresponding author on reasonable request.
